# Cellular magnetic resonance imaging contrast generated by the ferritin heavy chain genetic reporter under the control of a Tet-On switch

**DOI:** 10.1186/s13287-015-0205-z

**Published:** 2015-10-31

**Authors:** Xiaoya He, Jinhua Cai, Bo Liu, Yi Zhong, Yong Qin

**Affiliations:** Department of Radiology, Children’s Hospital of Chongqing Medical University, 136 Zhongshan 2 Road, Yuzhong District, Chongqing, 400014 China; Ministry of Education Key Laboratory of Child Development and Disorders, 136 Zhongshan 2 Road, Yuzhong District, Chongqing, 400014 China; Key Laboratory of Pediatrics in Chongqing, 136 Zhongshan 2 Road, Yuzhong District, Chongqing, 400014 China; Chongqing International Science and Technology Cooperation Center For Child Development and Disorders, 136 Zhongshan 2 Road, Yuzhong District, Chongqing, 400014 China

**Keywords:** Magnetic resonance imaging, Ferritin heavy chain, Reporter gene, Stem cells, Tetracycline inducible expression system

## Abstract

**Introduction:**

Despite the strong appeal of ferritin as a magnetic resonance imaging (MRI) reporter for stem cell research, no attempts have been made to apply this genetic imaging reporter in stem cells in an inducible manner, which is important for minimizing the potential risk related to the constitutive expression of an imaging reporter. The aim of the present study was to develop an inducible genetic MRI reporter system that enables the production of intracellular MRI contrast as needed.

**Methods:**

Ferritin heavy chain (FTH1) was genetically modified by adding a Tet-On switch. A C3H10T1/2 cell line carrying Tet-FTH1 (C3H10T1/2-FTH1) was established via lentiviral transduction. The dose- and time-dependent expression of FTH1 in C3H10T1/2 cells was assessed by western blot and immunofluorescence staining. The induced “ON” and non-induced “OFF” expressions of FTH1 were detected using a 3.0 T MRI scanner. Iron accumulation in cells was analyzed by Prussian blue staining and transmission electron microscopy (TEM).

**Results:**

The expression of FTH1 was both dose- and time-dependently induced, and FTH1 expression peaked in response to induction with doxycycline (Dox) at 0.2 μg/ml for 72 h. The induced expression of FTH1 resulted in a significant increase in the transverse relaxation rate of C3H10T1/2-FTH1 cells following iron supplementation. Prussian blue staining and TEM revealed extensive iron accumulation in C3H10T1/2–FTH1 cells in the presence of Dox.

**Conclusions:**

Cellular MRI contrast can be produced as needed via the expression of FTH1 under the control of a Tet-On switch. This finding could lay the groundwork for the use of FTH1 to track stem cells *in vivo* in an inducible manner.

## Introduction

Stem cells, which exhibit the capacity to differentiate into a diverse range of specialized cell types, have been widely applied in the treatment of human diseases, including cardiovascular, bone degenerative, autoimmune, and neurodegenerative diseases [1–6]. Along with the development of stem cell therapy for clinical practice, it is crucial to visualize and track cells efficiently to evaluate and improve this therapeutic approach [1, 7]. Traditional histopathologic methods, which require repeated biopsies or euthanasia and the removal of tissue for cell detection in animal studies, cannot be completely applied to patients [7, 8]. Therefore, there is an urgent need to develop noninvasive imaging tools that enable the longitudinal assessment of the viability, proliferation, lineage differentiation, and functional integration of cell grafts.

Several imaging techniques have been investigated for monitoring grafted cells *in vivo*. Optical imaging techniques, such as bioluminescence imaging [9] and fluorescence imaging [10], have been used to track cell grafts in small animals. However, optical imaging shows that the sensitivity and spatial resolution deteriorate more rapidly in deeper tissues due to light scattering, which is not feasible in large animals [3, 8, 11]. The imaging of radionuclide-labeled cells using single-photon emission computed tomography (SPECT) or positron emission tomography (PET) has also been applied to track stem cell grafts. One serious disadvantage of this technique is that PET and, particularly, SPECT have inferior spatial resolution [12]. In addition, frequent injections of radioactive tracers are needed to obtain timely data about the cell grafts [3]. Compared with radionuclide-based or optical imaging, magnetic resonance imaging (MRI) has been proposed as an ideal technique to monitor cells because of its high spatial resolution and soft tissue contrast [1, 6, 13].

To date, MR imaging of stem cells has relied on the cellular contrast generated by two major principles: direct labeling and indirect labeling (based on MRI reporter gene expression) [14, 15]. Direct cell labeling refers to the strategy of loading cells with contrast agents prior to engraftment into the host. Due to their high relaxivity, superparamagnetic iron oxide nanoparticles (SPIOs) have been the most widely used contrast agents for tracking various cell types [3, 12, 15–18]. However, long-term tracking of engrafted cells is not possible when using this direct labeling approach because the concentration of the contrast agent in the cells continuously decreases as they divide [16, 19, 20]. Moreover, the MRI signal produced by these agents does not reflect the actual cell number because the cells can continue to proliferate and divide asymmetrically [3, 21]. In addition, the MRI signal from magnetic nanoparticles does not represent the number of live cells because particles released from dead cells can be phagocytosed by host cells, thereby misleading MRI tracking [2, 22, 23]. To circumvent these shortcomings, it is necessary to use a genetic MRI reporter system, in which the cells to be implanted are transfected with reporter genes that are integrated into the genome, expressed in a sustainable manner, and ultimately translated into traceable products, resulting in MRI contrast in the transplanted cells.

Several genetic MRI reporting systems have been investigated, including divalent metal transporters [24, 25], β-galactosidase [26], tyrosinase [27], transferrin receptor [28], magA [29, 30] and ferritin [31, 32]. Among these candidates, ferritin, which displays a high transverse relaxation rate by inducing the expression of the transferrin receptor and increasing iron uptake, has been widely used as a reproducible and functional MRI reporter in extensive molecular imaging studies [2, 33]. Ferritin is a ubiquitous intracellular iron storage protein consisting of 24 heavy (H) and light (L) subunits, and it is essential for cellular iron metabolism [19, 34, 35]. One candidate major regulator of ferritin activity was identified as ferritin heavy chain (FTH1), which displays ferroxidase activity to promote iron oxidation and incorporation [36]. FTH1 alone or in conjunction with ferritin light chain has been previously reported to function as an MRI reporter [37, 38]. In contrast to SPIOs and other particles, the genetically modified transgene of FTH1 would not be diluted as cells divide. In this regard, the continuous production of FTH1 in the daughter cells offers a significant advantage for cell tracking via MRI compared with particle-based cell labeling methods [2, 39]. Thus far, few studies have focused on FTH1 as an emerging reporter. These studies showed that the overexpression of FTH1 alters the MRI signal at its expression site and enables cell tracking via MRI.

Despite the strong appeal of FTH1 as an MRI reporter for stem cell research, no attempts have been made to apply this imaging reporter in stem cells in an inducible manner, which is important for minimizing the potential toxicity caused by the constitutive expression of the imaging reporter. In our study, C3H10T1/2 stem cells were transduced with FTH1 as a MRI reporter, which was regulated by a Tet-On switch. Our aim was to minimize the potential risk of the persistent overexpression of the reporter gene and to monitor stem cells via MRI as needed.

## Methods

### Cell preparation

Wild type C3H10T1/2 cells (C3H10T1/2-WT; kindly gifted by the Molecular Cancer Laboratory of the University of Chicago in the United States), a murine mesenchymal stem cell line, were grown in Dulbecco’s Modified Eagle’s Medium (DMEM; Gibco, Grand Island, NY, USA) supplemented with 10 % fetal bovine serum (FBS; Hyclone, Logan, UT, USA) and 0.5 % streptomycin at 37°C in 5 % CO_2_. The medium was changed daily, and the cells were passaged every three days at a ratio of 1:3 to 1:5 by treating the cells with 0.25 % trypsin-EDTA (Beijing Dingguo Biological Technology Co., Ltd., Beijing, China), followed by dissociation into single cells.

### Construction and transfection of recombinant lentiviral vectors

To generate recombinant lentiviruses expressing a fusion gene of FTH1 with a Flag tag under the control of a tetracycline response element (TRE), human FTH1 (accession number BC000857) cDNA was generated via polymerase chain reaction (PCR) amplification using the following primers: forward, AACCGTCAGATCGCACCGGTGCCACCATGACGACCGCGTCCACCTC; reverse, TCCTTGTAGTCCATGAATTCGCTTTCATTATCACTGTCTC. Then, this cDNA was subcloned into the multiple cloning site of the pLenti-Tet-on-MCS-3Flag-Puro plasmid using *AgeI* and *EcoRI* to yield the recombinant vector pLenti-Tet-on-FTH1-3Flag-Puro (pLV-Tet-FTH1). The production of pLV-Tet-FTH1 was verified by PCR analysis and DNA sequencing. A lentivirus expressing Tet-FTH1 (LV-Tet-FTH1) was generated by co-transfecting pLV-Tet-FTH1 together with the packaging vector pHelper 1.0 and the envelope vector pHelper 2.0 into 293 T packaging cells (Invitrogen, Carlsbad, CA, USA). Fresh medium containing 10 % FBS was added 10-14 h after transfection, and the viral medium was collected at 48–72 h. C3H10T1/2 cells were infected with the lentiviruses at 30–40 % confluence using polybrene (8 μg/ml) (Sigma-Aldrich, St. Louis, MO, USA). A t 72 h post-transduction, the medium was supplemented with 4 μg/ml puromycin (Sigma-Aldrich, St. Louis, MO, USA) for selection to generate a clonal cell line (C3H10T1/2-FTH1).

### Western blot analysis

To examine the dose-dependent expression of FTH1, C3H10T1/2-FTH1 cells were cultured in medium containing doxycycline (Dox; Santa Cruz, Dallas, TX, USA) at serial concentrations (0, 0.02, 0.2, 0.6, 2, or 6 μg/ml) for 72 h. Then, the time point of peak FTH1 expression was determined by culturing the cells in medium containing the optimal concentration of Dox for different durations. After treatment, the cells were washed with ice-cold phosphate-buffered saline (PBS, pH 7.4) and lysed in radioimmunoprecipitation assay (RIPA) buffer (Beyotime, Nanjing, Jiangsu, China). The lysates were heated at 100 °C for ten min and clarified by centrifugation at 14,000 × rpm at 4 °C for 15 min. The total protein concentration was determined using bicinchoninic acid (BCA; Beyotime, Nanjing, Jiangsu, China) method. A total of 30 μg of protein was separated via 12 % gradient SDS-polyacrylamide gel electrophoresis (SDS-PAGE; Beyotime, Nanjing, Jiangsu, China) and transferred to polyvinylidene fluoride (PVDF) membranes (Millipore, Madrid, Spain), which were then blocked with 5 % bovine serum albumin (BSA, Sigma-Aldrich, St. Louis, MO, USA) in Tris-buffered saline containing Tween-20 (TBST). The membranes were probed with primary antibodies that specifically recognized FTH1 (rabbit anti-FTH1, 1:1,000; Abcam, Cambridge, MA, UK) or β-actin (mouse anti-β-actin; Nanjing Zoonbino Biotechnology Co., Ltd., Nanjing, Jiangsu, China) overnight at 4 °C. After washing several times, the membranes were incubated with secondary antibodies (anti-rabbit 1:5,000, Abgent, San Diego, CA, USA; anti-mouse 1:1000, Genscript, Nanjing, Jiangsu, China) and visualized using an enhanced chemiluminescence kit (Beyotime, Nanjing, Jiangsu, China). FTH1 expression was quantified and normalized to β-actin expression using Quantity One 4.4 software (Bio-Rad, Hercules, CA, USA).

### Immunofluorescence staining of cells

The Flag tag was used to indirectly determine the expression levels of FTH1 via immunocytochemistry using a Flag-specific antibody. C3H10T1/2-FTH1 cells were cultured for 72 h in the same concentrations of Dox as those used in the western blot experiments. Then, the cells were fixed in 4 % paraformaldehyde (PFA) for 15 min at room temperature. The fixative solution was removed, and the cells were washed with PBS three times for five min each. The cells were permeabilized with 1 % Triton X-100 in PBS for ten min, blocked with 5 % BSA in PBS at 37 °C for 30 min to 1 h, and then incubated in a specific primary antibody (mouse anti-Flag 1:200; Sigma-Aldrich, St. Louis, MO, USA) overnight at 4 °C. After three washes with PBS, the cells were incubated in a secondary antibody (Cy3-conjugated anti-mouse, 1:1,000; Beyotime, Nanjing, Jiangsu, China) for 30-45 min at 37 °C. Nuclei were counterstained with 4,6-diamidino-2-phenylindole (DAPI; Beyotime, Nanjing, Jiangsu, China) for five min. Images were acquired using a biological fluorescence microscope (Nikon, Tokyo, Japan).

### Cellular MRI

To determine the suitable concentration of iron supplementation to produce cellular MRI contrast in the presence of FTH1 expression, C3H10T1/2-FTH1 cells were cultured in varying concentrations of ferric ammonium citrate (FAC; Sigma-Aldrich, St. Louis, MO, USA) in the presence or absence of 0.2 μg/ml Dox for 72 h. Then, the following six treatments of C3H10T1/2-FTH1 cells were prepared: (1) no Dox or FAC; (2) no Dox and 500 μM FAC; (3) 0.2 μg/ml Dox and no FAC; (4) 0.2 μg/ml Dox and 500 μM FAC; (5) 6 μg/ml Dox and no FAC; and (6) 6 μg/ml Dox and 500 μM FAC. After treatment for 72 h, the cells were thoroughly washed and dissociated with trypsin, followed by fixation in 4 % PFA for 15 min. Each group of cells (3-5 × 10^7^ cells) was centrifuged at 1,000 rpm for five min, and the supernatant was removed. Then, the cells were resuspended in 100 μl of PBS and carefully placed in 0.5-ml Eppendorf tubes. The cells settled and formed a loose pellet at the bottom of the tubes. All MRI scans were performed using a clinical 3.0 T MRI scanner (Phillips, Eindhoven, Netherlands). The multi-slice and multi-echo sequence was used for T_2_ mapping *in vitro*. The imaging parameters were as follows: time of repetition (TR) = 2,000 ms, time of echo (TE) = 13-78 ms with a step size of 13 ms (six-point T_2_ mapping), field of view (FOV) = 160 × 160 mm, image matrix = 380 × 311, and slice thickness = 1 mm. R_2_ values were measured from R_2_ color maps, which were merged with the anatomical images acquired at the shortest TE.

### Prussian blue staining of cells

Prussian blue staining was used to assess iron accumulation in stem cells. C3H10T1/2-WT or C3H10T1/2-FTH1 cells cultured on poly-L-lysine-coated coverslips were treated as follows: no Dox or FAC (Dox-/FAC-); no Dox and 500 μM FAC (Dox-/FAC 500); or 0.2 μg/ml Dox and 500 μM FAC (Dox 0.2/FAC 500). After incubation for 72 h, the cells were washed thoroughly in PBS and fixed in 4 % PFA for 15 min before staining. The coverslips were placed in staining solution (2 % potassium ferrocyanide and 2 % HCl) for 30 min at room temperature. After three washes with PBS, the cells were counterstained with nuclear fast red and mounted on slides using water-soluble mounting medium. In this assay, the presence of iron was indicated as a bright blue color displaying a granular cytoplasmic distribution.

### Transmission electron microscopy of cells

C3H10T1/2-WT and C3H10T1/2-FTH1 cells were treated as described for Prussian blue staining. At least 1.5 × 10^6^ cells were fixed in 3 % glutaraldehyde-cacodylate buffer at 4 °C overnight. After incubation for 1 h in 1 % OsO_4_, the cells were dehydrated in a graded ethanol series and embedded in artificial Epon resin (Hexion, Shanghai, China). Unstained thin sections of the cells were evaluated for FAC endocytosis using a H-7500 transmission electron microscope (Hitachi, Tokyo, Japan).

### Assessment of cell proliferation

To assess whether FTH1 expression or FAC is detrimental to cell growth, 5 × 10^3^ C3H10T1/2-WT and C3H10T1/2-FTH1 cells per well treated with 0.2 μg/ml Dox, 500 μM FAC, or both were plated in a 96-well plate and incubated for 72 h. Then, cell proliferation was measured via a Cell Counting Kit-8 (CCK-8) assay according to the manufacturer's instructions (Beyotime). To further address the adverse impact of Dox on cell growth, the cells were treated with varying concentrations of Dox for 72 h, and cell proliferation was determined using the same assay. Changes relative to the untreated group were calculated for both cell lines. The absorbance was measured at a wavelength of 450 nm, and these values were subtracted from the absorbance values at the reference wavelength of 650 nm.

### Statistical analysis

All data are expressed as the means ± SD of at least five independent experiments. Statistical analyses were completed using a two-tailed unpaired Student’s *t*-test. *P*-values less than 0.05 were considered statistically significant.

## Results

### Expression of FTH1 induced by Dox in C3H10T1/2-FTH1 cells

**Fig. 1:**
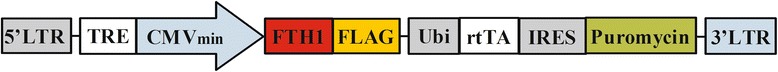
Schematic illustration of LV-Tet-FTH1. A flag tag was fused to the 3’ end of the FTH1 gene. The expression of Flag-tagged FTH1 was under the control of a tetracycline response element (*TRE*). The antibiotic resistance gene puromycin was expressed via the internal ribosome entry site (*IRES*) downstream of reverse tetracycline-controlled transactivator (*rtTA*) and was regulated by the human ubiquitin (*Ubi*) promoter. *LV-Tet-FTH1* lentiviruses expressing Tet-FTH1 *FTH1* ferritin heavy chain

To express FTH1 only at the time of MRI performance, the FTH1 gene was cloned into a Dox-inducible lentiviral vector, and a Flag tag was fused to the 3’ end of the FTH1 gene. Then, the expression of FTH1 carrying the Flag tag was under the control of a Tet-On switch. Puromycin, an antibiotic-resistant gene, was expressed via the internal ribosome entry site (IRES) downstream of reverse tetracycline-controlled transactivator (rtTA) and was regulated by the human ubiquitin (Ubi) promoter. The resulting lentiviral vector carrying FTH1 under the control of a Tet-On switch (LV-Tet-FTH1) is illustrated in Fig. 1. This high-titer lentivirus, which was prepared as described above, was used to transfect C3H10T1/2 cells, followed by clonal selection using puromycin. Then, C3H10T1/2 cells expressing Tet-FTH1 (C3H10T1/2-FTH1) were successfully established for subsequent studies.

**Fig. 2:**
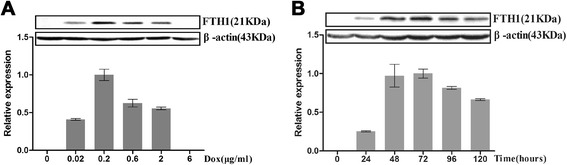
Western blot assay of doxycycline-regulated FTH1 expression. **a** Dox dose-dependent FTH1 expression: C3H10T1/2-FTH1 cells were cultured in medium containing Dox at different concentrations for 72 h. With increasing Dox concentration, the FTH1 expression level increased, peaked at a Dox concentration 0.2 μg/ml, and then decreased at higher Dox concentrations. FTH1 expression was completely repressed at a Dox concentration of 6 μg/ml. **b** Time-dependent FTH1 expression: C3H10T1/2-FTH1 cells were cultured in medium containing Dox (0.2 μg/ml) for different durations. With increasing time, the FTH1 expression level increased gradually to its peak at 72 h and then decreased gradually. *FTH1* ferritin heavy chain, *Dox* doxycycline

**Fig. 3:**
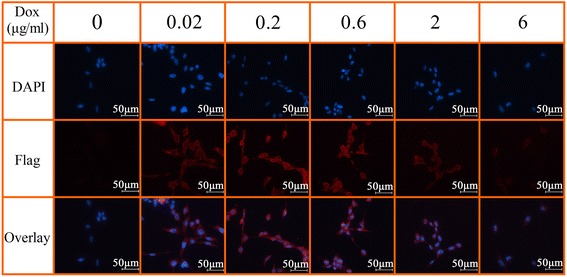
Immunostaining of doxycycline-regulated Flag expression in C3H10T1/2-FTH1 cells. The cells were grown in different concentrations of Dox for 72 h. Immunostaining using a Flag-specific antibody demonstrated that with increasing Dox concentration, the expression of Flag in C3H10T1/2-FTH1 cells increased and peaked upon induction with 0.2 μg/ml Dox for 72 h and then decreased (*red*); this result is consistent with the western blot results. The nucleus was visualized via DAPI staining (*blue*). Bar = 50 μm. *FTH1* ferritin heavy chain, *Dox* doxycycline, *DAPI* 4,6-diamidino-2-phenylindole

To examine the dose-dependent expression of FTH1, C3H10T1/2-FTH1 cells were cultured in medium containing Dox at different concentrations for 72 h, and different levels of FTH1 expression were detected by western blot analysis. FTH1 expression peaked at a Dox concentration of 0.2 μg/ml and decreased at higher Dox concentrations. FTH1 expression was completely repressed at a Dox concentration of 6 μg/ml (Fig. 2a). For the time-dependent expression of FTH1, C3H10T1/2-FTH1 cells in the absence of Dox were transferred to medium containing 0.2 μg/ml Dox and incubated for different durations. The results showed that the FTH1 expression level increased gradually to its peak at 72 h and then decreased gradually (Fig. 2b). Furthermore, immunostaining using a Flag-specific antibody demonstrated that Flag expression peaked in C3H10T1/2-FTH1 cells upon induction with 0.2 μg/ml Dox for 72 h (Fig. 3); this result is consistent with the western blot results. Neither FTH1 nor Flag expression was detectable in the absence of Dox. Based on protein expression, a Dox concentration of 0.2 μg/ml and a duration of 72 h were used as the optimal induction conditions to achieve maximal FTH1 expression in subsequent studies.

### MRI contrast generated via the induced expression of FTH1

**Fig. 4:**
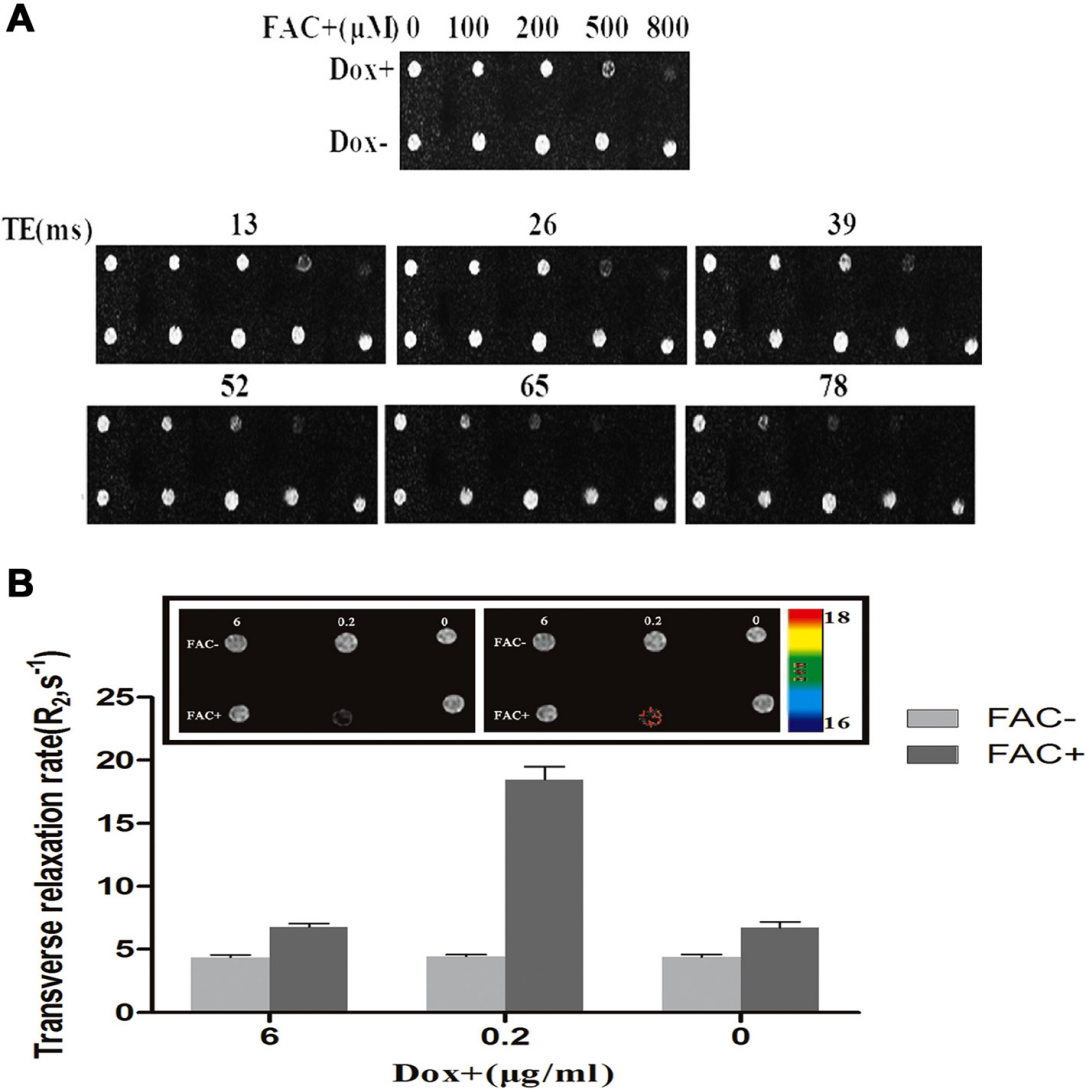
MRI contrast generated via the induced expression of FTH1. **a** C3H10T1/2-FTH1 cells were cultured for 72 h in the presence or absence of 0.2 μg/ml Dox and different concentrations of FAC for T_2_-weighted imaging. In the absence of Dox (Dox-), there was no significant MRI signal change. However, in the presence of Dox (Dox+), the MRI signal decreased with increasing iron concentrations, and the MRI signal was significantly decreased at iron concentrations of 500 μM and 800 μM. **b** C3H10T1/2-FTH1 cells were cultured in Dox at different concentrations in the presence or absence of 500 μM FAC for 72 h. Color-coded R_2_ maps from multi-echo measurements of the R_2_ relaxation rates showed a significant increase in the R_2_ rate in C3H10T1/2-FTH1 cells treated with Dox and FAC (0.2 μg/ml and 500 μM, respectively) compared to that in the other cells (*P* < 0.05). *MRI* magnetic resonance imaging, *FTH1* ferritin heavy chain, *Dox* doxycycline, *FAC* ferric ammonium citrate

The results showed no significant changes in the MRI signal in the absence of Dox (Dox-); however, there was a significant decrease in the MRI signal in the presence of Dox (Dox+) and 500 μM FAC. Although further increasing the FAC concentration additionally reduced the MRI signal, 500 μM FAC was used in the subsequent *in vitro* MRI experiments considering the side effects of a high FAC concentration on the cells (Fig. 4a). We further measured the changes in R_2_ in C3H10T1/2-FTH1 cells incubated in Dox at different concentrations in the presence or absence of 500 μM FAC for 72 h. The results demonstrated a marked increase in *R*_2_ in C3H10T1/2-FTH1 cells treated with Dox and FAC (0.2 μg/ml and 500 μM, respectively) compared with that in the other cells (*P* < 0.05) (Fig. 4b). These results indicated that the resulting MRI contrast could be correlated with gene expression via Dox administration and iron supplementation.

### Evaluation of cell proliferation

**Fig. 5:**
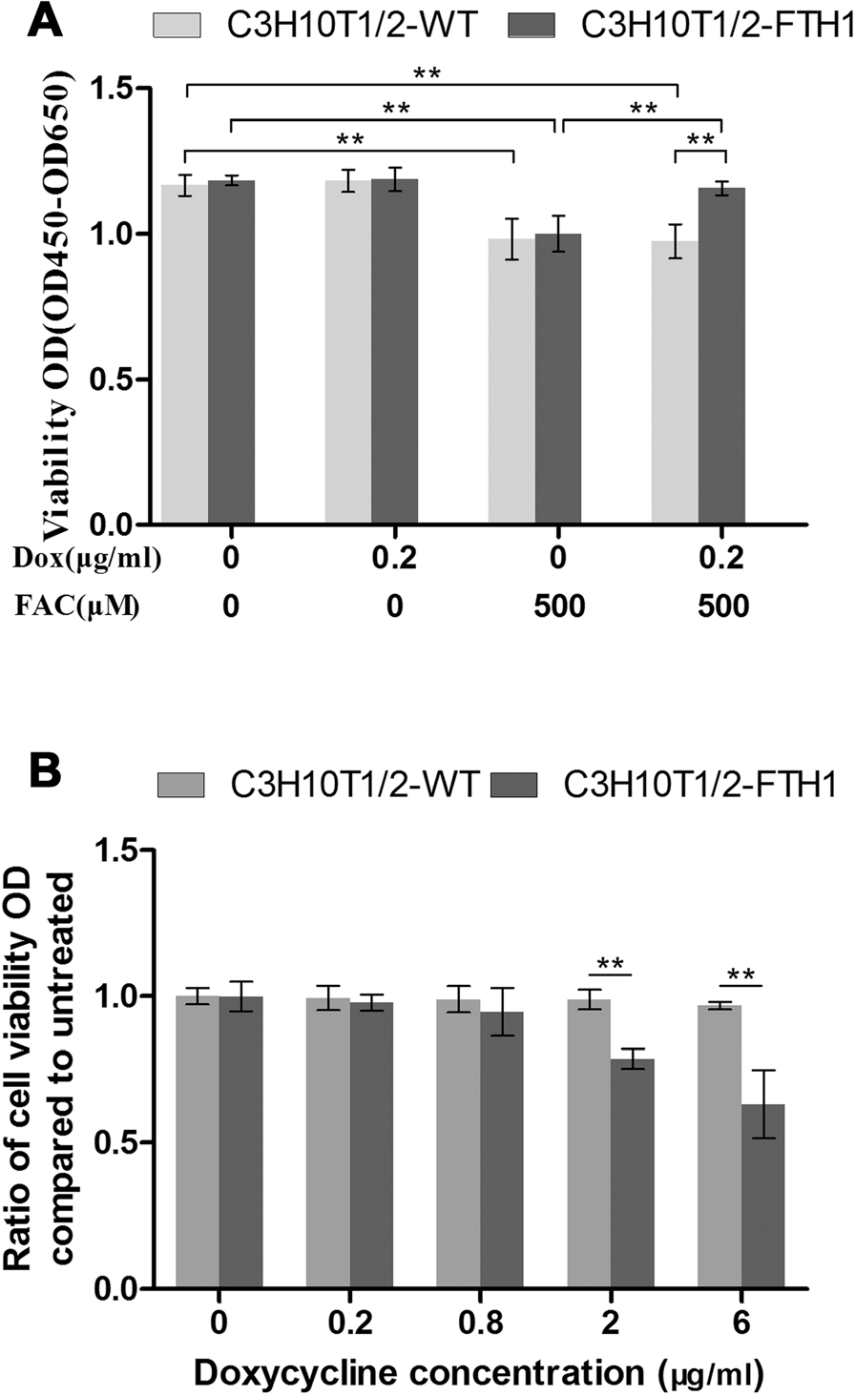
Evaluation of cell proliferation. **a** To determine the impact of FTH1 expression on cells, the proliferation of treated cells (5 × 10^3^) cultured in a 96-well plate for 72 h was assessed using Cell Counting Kit-8 (CCK-8). A high dose of FAC (500 μM) inhibited the growth of both C3H10T1/2-WT cells in the presence or absence of Dox and C3H10T1/2-FTH1 cells only in the absence of Dox (*P* < 0.05). However, maximal FTH1 expression did not interfere with C3H10T1/2 cell proliferation *in vitro*, even in the presence of iron supplementation. **b** The impact of Dox on cell growth was also determined using CCK-8. There was no significant suppression of cell growth at a Dox concentration of 0.8 μg/ml. However, significant suppression of the growth of C3H10T1/2-FTH1 cells compared with C3H10T1/2-WT cells was detected at a Dox concentration of 2 μg/ml (*P* < 0.001, n = 5). At higher concentrations of Dox, the proliferation of both C3H10T1/2-WT and C3H10T1/2-FTH1 cells negatively correlated with the Dox concentration, although the C3H10T1/2-FTH1 cells showed a slightly steeper negative linear correlation than did the C3H10T1/2-WT cells. *FTH1* ferritin heavy chain, *FAC* ferric ammonium citrate, *Dox* doxycycline

To determine the impact of expressing FTH1 on cells, we evaluated the cell proliferation rate via the CCK-8 assay. The results revealed that maximal FTH1 expression did not interfere with C3H10T1/2 cell proliferation *in vitro*, even in the presence of iron supplementation. However, we observed that supplementation of the cell medium with FAC at a high dose (500 μM) inhibited the growth of both C3H10T1/2-WT cells in the presence or absence of Dox and C3H10T1/2-FTH1 cells only in the absence of Dox (*P* < 0.05). These observations indicated that the high iron concentration was toxic to the wild type cells but not the cells overexpressing FTH1 (Fig. 5a). Supplementation of the cell medium with a low concentration of FAC (5 μM) did not affect wild type or transduced cell growth in the presence or absence of 0.2 μg/ml Dox (data not shown). In summary, in our study, FTH1 overexpression did not affect C3H10T1/2 cell proliferation, and FTH1 increased the resistance to iron toxicity. We further addressed the side effects of Dox on cell growth using the same method. The growth rate of C3H10T1/2-FTH1 cells was not significantly different from that of C3H10T1/2-WT cells at corresponding Dox concentrations of 0.2 μg/ml or 0.8 μg/ml. However, C3H10T1/2-FTH1 cells demonstrated a significantly lower growth rate than did C3H10T1/2-WT cells at a Dox concentration of 2 μg/ml (*P* < 0.001, n = 5). At higher Dox concentrations, the growth rate of both C3H10T1/2-FTH1 and C3H10T1/2-WT cells negatively correlated with the Dox concentration, although C3H10T1/2-FTH1 cells were more sensitive to these concentrations of Dox than were C3H10T1/2-WT cells (Fig. 5b).

### Iron accumulation in C3H10T1/2 cells following the induced expression of FTH1

**Fig. 6:**
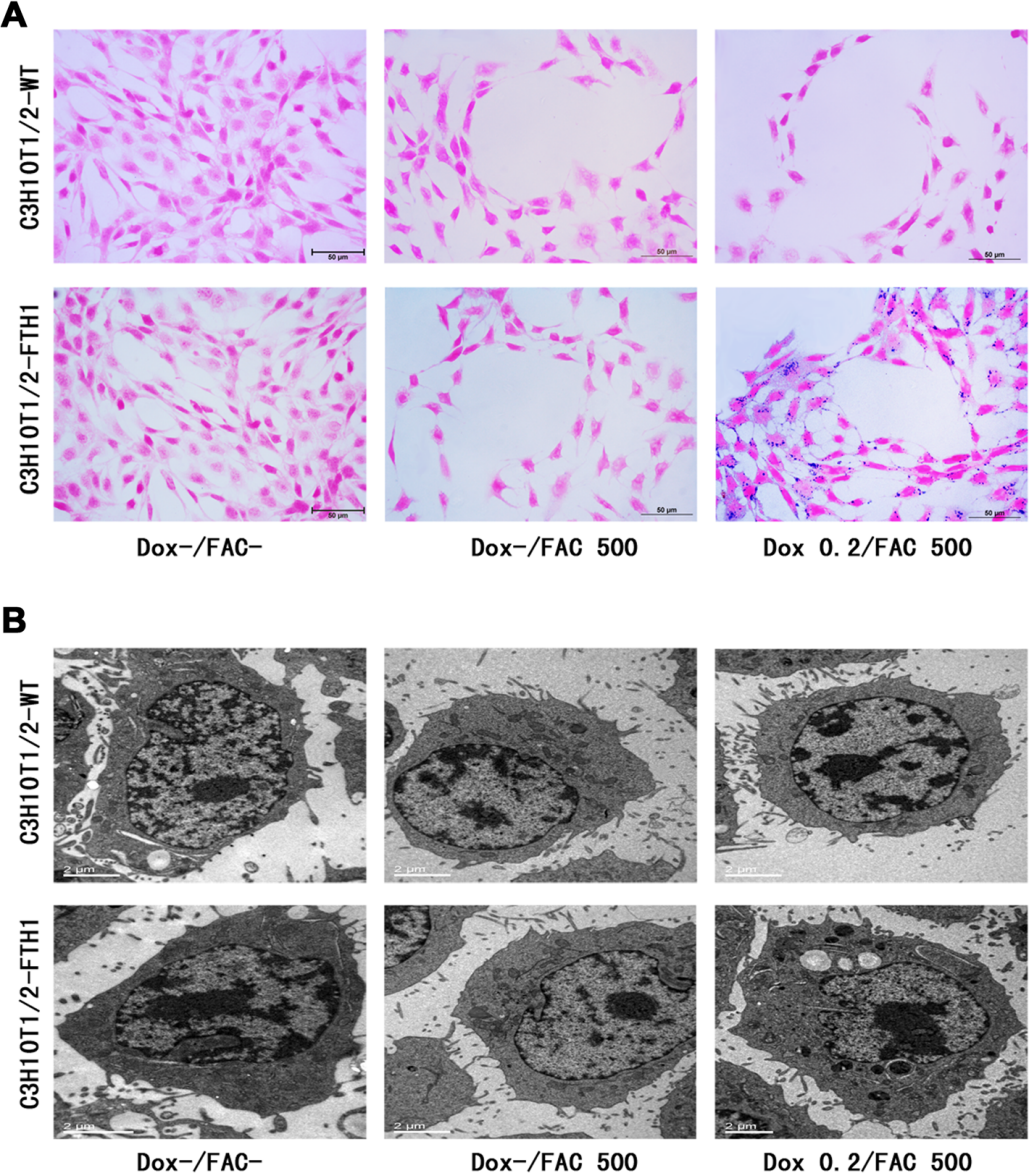
Intracellular iron accumulation following induced FTH1 expression. **a** Prussian blue staining: In the presence of 0.2 μg/ml Dox and 500 μM FAC, a large number of *blue* iron particles were detected in the C3H10T1/2-FTH1 cells, but no iron particles were detected in the C3H10T1/2-WT cells. In the absence of Dox, neither C3H10T1/2-FTH1 nor C3H10T1/2-WT cells showed *blue* iron particles, regardless of FAC supplementation. **b** Transmission electron microscopy: In the presence of 0.2 μg/ml Dox and 500 μM FAC, numerous electron dense particles were detected in the cytoplasm of C3H10T1/2-FTH1 cells, but no particles were detected in C3H10T1/2-WT cells. In the absence of Dox, neither C3H10T1/2-FTH1 nor C3H10T1/2-WT cells showed electron dense particles, regardless of FAC supplementation. Scale bars: 50 μm (**a**) and 2 μm (**b**). *FTH1* ferritin heavy chain, *Dox* doxycycline, *FAC* ferric ammonium citrate

Prussian blue staining confirmed more significant accumulation of iron in the C3H10T1/2-FTH1 cells than in the C3H10T1/2-WT cells in the same Dox-/FAC-supplemented medium (0.2 μg/ml Dox and 500 μM FAC). In the absence of Dox, neither C3H10T1/2-FTH1 nor C3H10T1/2-WT cells showed obvious blue staining particles regardless of FAC supplementation (Fig. 6a). TEM revealed results similar to those of Prussian blue staining (Fig. 6b). Extensive iron particles accumulated in the cytoplasmic vacuoles of C3H10T1/2-FTH1 cells cultured in the presence of 0.2 μg/ml Dox and 500 μM FAC. However, the cells (C3H10T1/2-WT and C3H10T1/2-FTH1) cultured in control medium did not show any iron accumulation. These results indicated that FTH1 overexpression may trigger an increase in the capacity of the cell to internalize and store iron when iron is available.

## Discussion

Using an inducible Tet-On system to express the genetic MRI reporter FTH1, we successfully developed a noninvasive, repeatable and controllable imaging tool for stem cells. By employing this newly created system, we visualized these stem cells via MRI in a controlled manner during the Dox induction time window. More importantly, this Tet-On switch-controlled genetic MRI reporter system efficiently reduced the potential risk caused by the continuous expression of FTH1 and the accumulation of iron in stem cells.

The potential adverse impacts of persistent FTH1 expression and iron accumulation on cellular iron homeostasis and cell growth warrant concern. In our study, a 500 μM concentration of iron supplement reduced the growth rate of both C3H10T1/2-WT and C3H10T1/2-FTH1 stem cells in the absence of Dox (i.e., no FTH1 expression) but did not reduce the growth rate of C3H10T1/2-FTH1 stem cells in the presence of Dox (i.e., during FTH1 expression). This finding indicated that FTH1 expression can protect against reactive oxygen species, possibly via a mechanism involving the sequestering of excess iron [40]. These results agree with the study by Naumova et al. [2], in which the supplementation of the cell medium with FAC at a high dose (1 mM) inhibited the proliferation of wild type and transduced C2C12 cells; their study also revealed that exogenous iron was more toxic to wild type cells than to cells overexpressing ferritin. However, other authors have presented contrasting results. One study found that the growth of C6 glioma cells was significantly reduced when FTH1 expression was dramatically increased [16]. Another study indicated that the growth of HeLa cells was significantly decreased by the overexpression of FTH due to the induction of an iron-deficient phenotype and that HeLa cell growth was recovered by administering iron [41]. Additionally, Feng et al. [19] demonstrated that moderate overexpression of FTH1 did not affect nasopharyngeal carcinoma (NPC) cell growth with or without iron supplementation; moreover, the authors showed that the maximal overexpression of FTH1 decreased the cell growth rate in the absence of iron supplementation but that the cell growth rate remained unchanged in the presence of iron supplementation, which suggests that the expression level of FTH1 determined whether cell growth was affected. Based on the above evidence, we conclude that the effects of FTH1 may depend on the cell type and the FTH1 expression level.

In general, the accumulation of iron may have side effects in target cells because an excess of labile iron in cells is known to cause increased oxidative stress via Fenton and Haber–Weiss reactions, resulting in DNA, lipid, and protein damage [34]. Although FTH1 is considered a resistance factor to intracellular free iron, the intracellular free iron levels may exceed the sequestering capacity of persistently overexpressed FTH1. In addition, with iron transferred into the cells, the extracellular iron concentration could be reduced to varying degrees, thereby influencing iron homeostasis in the local tissue and even the entire body. We adopted an inducible Tet-On system in the present study, and we revealed that the expression of FTH1 could be controlled as needed. This method efficiently reduced the potential risk of persistent FTH1 overexpression and iron accumulation in cells, which is essential to the application of FTH1 as a MRI reporter for tracking transplanted cells *in vivo*.

The present study revealed that FTH1 expression was dose- and time-dependent upon Dox administration. In the dose-dependence experiment, western blot analysis showed no FTH1 expression in C3H10T1/2-FTH1 cells in the absence of Dox, indicating that there was no background leakage of gene expression in the tetracycline-regulated lentiviral vector system. At increasing Dox concentrations, FTH1 expression increased and peaked at 0.2 μg/ml Dox. Based on this result, the optimal incubation time of 72 h was obtained from the time-dependence experiment. In addition, immunostaining using a Flag-specific antibody demonstrated that the expression of Flag peaked in C3H10T1/2-FTH1 cells induced with 0.2 μg/ml Dox for 72 h. This finding was consistent with the western blot results. Thus, the expression of FTH1 in C3H10T1/2-FTH1 cells was efficiently switched on by adding a suitable dose of Dox for the appropriate duration. We further addressed the impact of Dox on cell growth. The proliferation rate of both C3H10T1/2-FTH1 and C3H10T1/2-WT cells negatively correlated with the Dox concentration, although C3H10T1/2-FTH1 cells were more sensitive to Dox than C3H10T1/2-WT cells. However, 0.2 μg/ml Dox, which resulted in maximal FTH1 expression, had a negligible impact on cell viability.

Notably, compared to *ex vivo* direct cell-labeling methods, such as using SPIOs, the genetic MRI reporter method provides much weaker MRI contrast [3, 16]. However, SPIOs can subsequently become diluted, thereby reducing their signal, due to cell division. Moreover, recent studies have shown that SPIOs may have an adverse impact on cell differentiation [42–44]. Thus, we expect that exogenous labeling methods might be helpful for monitoring differentiated cells, especially cells that exhibit a slow proliferation rate for a relatively short period. However, for tracking stem cells or progenitor cells with high proliferation and differentiation capacity, MRI reporters have a clear advantage over direct cell-labeling methods.

To date, a few MRI reporting systems have been investigated. Most of these genetic reporters were developed to track metastatic cancer cells, and only ferritin and magA have been reported with respect to monitoring stem cell grafts. Some studies have yielded evidence that the expression of magA can produce magnetite particles to augment MRI contrast [29, 45, 46]. Following magA expression, cell pellets consistently showed an increase in R_2_ of approximately three- to four-fold, compared with an increase in R_2_ of approximately 2.5-fold following ferritin expression [45]. However, in a recent study, Uebe et al. [47] indicated that magA is not involved in magnetosome formation; the authors proposed that the magnetosome gene island contains all essential genes required for magnetosome formation and that these genes might have been distributed by horizontal gene transfer. Compared with the pre-labeling approach and the use of other genetic MRI reporters described previously, FTH1 is the most attractive option for longitudinal MRI monitoring of molecular events. The results of the present study showed that the expression of FTH1 is tightly regulated by Dox in C3H10T1/2 stem cells. Using this inducible MRI reporter gene, the intracellular iron level was detectable using a clinical 3.0 T MRI system.

T_2_-weighted imaging presumably provides a more accurate representation of the spatial extent of the cells, although T_2_*-weighted imaging is more sensitive [45]. In our study, we were able to detect C3H10T1/2-FTH1 cells via T_2_-weighted imaging rather than T_2_*-weighted imaging. As expected, the R_2_ relaxation rate was significantly increased when C3H10T1/2-FTH1 cells were treated with Dox and FAC for the optimal period. Transverse relaxivity linearly increases with increasing magnetic field strength; therefore, we would expect more effective visualization of FTH1-tagged cells or grafts at higher magnetic field strengths.

Knowing the fate of the FTH1-tagged stem cells along the differentiation pathway is important. A recent study revealed that FTH1 does not alter the multidifferentiation potential of stem cells. FTH1 is expressed in differentiated cells as well as stem cells [48]. Thus, distinguishing between these cell types noninvasively is impossible via reporter gene imaging with the present available technology, and we cannot know whether the stem cells have differentiated into specific cell lines *in vivo*. In further studies, a novel imaging system should be developed to monitor and evaluate the differentiation of stem cells.

## Conclusions

In general, this study reports the first use of MRI for the detection of FTH1 gene expression in C3H10T1/2 stem cells in an inducible manner. In this study, we developed an inducible genetic MRI reporter system in which FTH1 is expressed under the control of a Dox-sensitive Tet-On switch. In C3H10T1/2 stem cells carrying this FTH1-Tet system, FTH1 expression was undetectable at the baseline but was strongly induced upon treatment with Dox. This increase in FTH1 expression resulted in substantial iron accumulation and clearly detectable MRI contrast in these cells. Potential future applications of this inducible MRI reporter system include tracking the transplanted stem cells by triggering reporter gene expression via the addition of a suitable dose of Dox only when MRI is needed. This method could efficiently reduce the potential risk caused by the persistent expression of this reporter gene and the constitutive accumulation of iron in cells.

## Abbreviations

CCK-8: Cell Counting Kit-8
Dox: Doxycycline
FAC: Ferric ammonium citrate
FTH1: Ferritin heavy chain
LV-Tet-FTH1: Lentiviruses expressing Tet-FTH1
MRI: Magnetic resonance imaging
PCR: Polymerase chain reaction
PET: Positron emission tomography
pLV-Tet-FTH1: pLenti-Tet-on-FTH1-3Flag-Puro
R_2_: Transverse relaxation rate
SPECT: Single-photon emission computed tomography
SPIOs: Superparamagnetic iron oxide nanoparticles
TEM: Transmission electron microscopy
Tet: Tetracycline
